# Analysis of Nematode Ventral Nerve Cords Suggests Multiple Instances of Evolutionary Changes to Neuron Number

**DOI:** 10.1111/ede.70037

**Published:** 2026-04-05

**Authors:** Jaeyeong Han, Alyson Ficca, Marissa Lanzatella, Kanika Leang, Matthew Barnum, Jonathan C. T. Boudreaux, Nathan E. Schroeder

**Affiliations:** ^1^ Department of Crop Sciences University of Illinois Urbana Illinois USA; ^2^ School of Integrative Biology University of Illinois Urbana Illinois USA; ^3^ Department of Biochemistry University of Illinois Urbana Illinois USA

**Keywords:** ecdysozoan, Mononchidae, motorneuron, sensory neuron

## Abstract

Despite their diversity in habitats, nematodes are often considered to have a highly conserved neuroanatomy. This premise is based on only a subset of the nematode phylogenetic tree within the subclass Chromadoria, which includes the model organism *Caenorhabditis elegans*, thereby limiting our understanding of macroevolutionary trends in nervous system structure. To approach this problem, we used nuclear morphology to quantify the number of neurons in the nematode ventral nerve cord (VNC) across the phylum and identified evolutionary patterns in neuroanatomical organization. Nuclear staining revealed that Dorylaimia has significantly more VNC neuronal nuclei than other taxa in Enoplia and Chromadoria, with some species having four times the number of neurons as *C. elegans*. These results suggest at least two independent transitions in VNC neuron number across subclasses. To further examine developmental patterns and potential variation in nervous system architecture of species with substantially more neurons than *C. elegans*, we established an isogenic culture of *Mononchus aquaticus* (Dorylaimia). We found that while *M. aquaticus* contained four times as many VNC neuronal nuclei as *C. elega*ns, the VNC had a similar developmental timeline during post‐embryonic stages. However, dye‐filling assays also revealed an extensive distribution of neurons along the lateral body wall of *M. aquaticus*, which have no obvious homologs in *C. elegans*. We further found that *M. aquaticus* is capable of sustained movement following bisection and speculate that this ability results from a more decentralized neuronal network. Our results provide a roadmap for understanding phylum‐wide nervous system evolution and demonstrate large‐scale differences in neuroanatomy across the phylum.

## Summary

Nematode neuroanatomy has undergone multiple large‐scale changes in neuron number across subclasses. The predatory nematode, *Mononchus aquaticus*, has a more extensive nervous system, including body wall neurons with no obvious homologs, compared with *Caenorhabditis elegans*.

## Graphical abstract

“Analysis of Nematode Ventral Nerve Cords Suggests Multiple Instances of Evolutionary Addition and Loss of Neurons” Han et al. DAPI staining of *Xiphinema* sp. (Clade 2) highlighting the neuronal‐like nuclei within the ventral nerve cord.

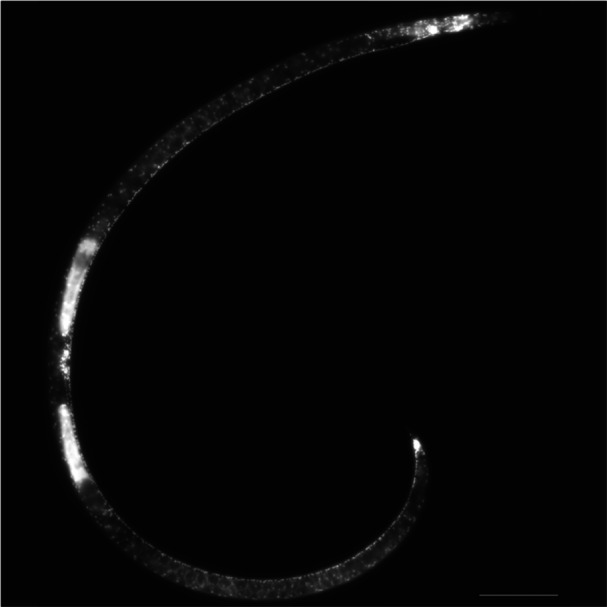

## 1 Introduction

Nematodes are among the most widespread and diverse animals on Earth, occupying a vast range of ecological niches. Despite their diversity, research on the nematode nervous system is dominated by studies of *Caenorhabditis elegans*. However, it seems unlikely that the *C. elegans* nervous system has been replicated exactly across the estimated 500 million plus years of nematode evolution (Qing et al. 2024). Recent years have seen an increased interest in understanding the nature and mechanisms of nervous system evolution in nematodes (Han et al. 2016; Toker et al. 2024; Wang et al. 2025). These studies point to distinct evolutionary changes, including the loss and gain of neurons, and set the stage for the use of nematodes to dissect broad principles of nervous system evolution. However, much of the current research focuses on only one part of the nematode tree.

**Figure 1:**
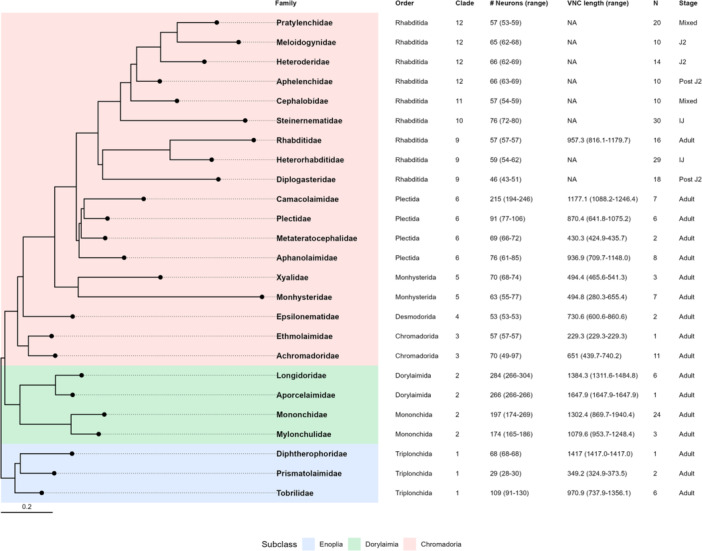
Phylogeny of nematode families used in this study with summary statistics for ventral nerve cord (VNC) neuronal nuclei. The tree was inferred using the maximum likelihood method and Tamura‐Nei model from SSU rDNA sequences obtained from Van Megen et al. (2009), including only families in this study. The number of neurons in clades 9–12 were obtained from previously published data (Han et al. 2016). Background colors indicate subclass: blue (Enoplia), green (Dorylaimia), and red (Chromadoria). The table provides order, clade (Van Megen et al. 2009), mean VNC neuronal nuclei count (# Neurons), mean VNC length in µm, sample size (*N*), and developmental stage. Scale bar: nucleotide substitutions per site. [Color figure can be viewed at wileyonlinelibrary.com]

Phylogenetic trees based on 18 s ribosomal DNA and genome sequences support the division of the phylum Nematoda into the subclasses Enoplia, Dorylaimia, and Chromadoria (Figure 1; Holterman et al. 2006; Qing et al. 2024; Van Megen et al. 2009) represented as either a five (Blaxter et al. 1998) or a 12 clade system (Holterman et al. 2006) adopted here. From these data, a consensus has emerged that the subclass Enoplia (Clade 1) is likely the earliest diverging nematode clade, followed by Dorylaimia (Clade 2). Nematodes in these earlier diverging clades differ from Chromadorian clades (Clades 3–12) in several aspects of their morphology and development (Schulze and Schierenberg 2011). For example, several species in Enoplia and Dorylaimia lack the deterministic development seen in embryonic development of *C. elegans* (Clade 9) and other species in more recently derived clades (Schulze and Schierenberg 2011).

The remarkable similarity in neuroanatomy between *C. elegans* and the gastrointestinal parasite *Ascaris suum* (Clade 8) suggests a high degree of nervous system conservation. Despite distinct habitats, sizes, and an estimated evolutionary separation of 350 million years, these species have nearly identical numbers (~300) of neurons (Qing et al. 2024; Stretton et al. 1978; Sulston 1976; White et al. 1976). Recent electron microscopy data comparing *C. elegans* to the satellite species *Pristionchus pacificus* (Clade 9) also found a nearly identical number of neurons in the anterior nervous system (Cook et al. 2025). However, these more detailed EM results also demonstrated instances of large‐scale rewiring of the nervous system (Bumbarger et al. 2013). Our previous light‐microscopy work compared nine nematode species within Clades 9–12 of the Chromadoria and found differences in the number of putative neurons within the ventral nerve cord (VNC), suggesting a previously unappreciated amount of neuroanatomy diversity among a subset of the phylum (Han et al. 2016). One limitation from these studies is that they excluded a large portion of the phylum including subclasses Enoplia and Dorylaimia and early‐branching groups in Chromadoria (Holterman et al. 2006).

Due to their relative recalcitrance to culturing, very few studies have examined the neuroanatomy of nematodes in those early‐diverging lineages. The handful of investigations suggested that certain nematodes in Enoplia and Dorylaimia may have an order of magnitude more neurons than found in *C. elegans* and other later diverging clades (Gans and Burr 1994; Malakhov 1994; Sulston and Horvitz 1977). These findings suggest a possible secondary simplification where the number of neurons decreased at some point during the evolution of the Chromadoria. To test the hypothesis of nervous system simplification, we used a survey approach to quantify the number of neurons in the VNC across Enoplia, Dorylaimia, and early diverging clades in Chromadoria (Clades 1–6 of Holterman et al. 2006). As this survey approach prevented examination of intraspecies variability and developmental timing, we also established an isogenic culture of *Mononchus aquaticus* (subclass Dorylaimia, Clade 2). By comparing our findings with existing knowledge on Chromadorian species such as *C. elegans*, we highlight both conserved and divergent neuroanatomy across the phylum.

## 2 Materials and Methods

### 2.1 Sample Collection and Nematode Extraction

Shallow aquatic sediment and terrestrial samples were collected and stored at 4°C until further processing. For aquatic samples, a liquid layer was always included to simulate the natural environment until processing. Most samples were collected from Champaign‐Urbana and surrounding areas in Illinois, with additional sites in Monmouth and Belleville, Illinois (Table 1). Marine samples were collected from the Carpinteria State Beach and Salt Marsh in California, courtesy of Drs. Tiago José Pereira and Holly Bik, formerly of the University of California, Riverside.

**Table 1:** List of sampling locations for the nematodes used for DAPI staining.

Sample location	Locality	GPS coordinates	Biome
Dana Colbert Park Pond	Savoy, IL	40°03′02.1″ N; 88°14′58.9″ W	Freshwater
Prairie Meadows Subdivision Pond	Savoy, IL	40°02′58.5″ N; 88°14′47.4″ W	Freshwater
Lake Park Residential Pond	Savoy, IL	40°03′53.2″ N; 88°14′21.1″ W	Freshwater
Moorman Sewage Retention Pond, Large (Lake Nalbandov)	Urbana, IL	40°05′22.2″ N; 88°14′08.4″ W	Freshwater
Moorman Sewage Retention Pond, Small (Lake Nalbandov)	Urbana, IL	40°05′16.5″ N; 88°14′11.3″ W	Freshwater
UIUC Japan House Main Pond	Urbana, IL	40°05′34.0″ N; 88°12′59.2″ W	Freshwater
UIUC Japan House Minor Pond	Urbana, IL	40°05′37.4″ N; 88°12'55.8″ W	Freshwater
Boneyard Creek	Champaign, IL	40°06′40.4″ N; 88°13′40.4″ W	Freshwater
Private Residence Pond	Urbana, IL	40°05′49.4″ N; 88°12′12.4″ W	Freshwater
Salt Fork River, Mulberry Tree	Homer, IL	40°05′39.1″ N; 87°99′65.4″ W	Terrestrial
Homer Lake Forest Preserve Collins Pond	Homer, IL	40°05′39.1″ N; 87°99′65.4″ W	Freshwater
Homer Lake Forest Preserve, White Oak	Homer, IL	40°05′39.1″ N; 87°99′65.4″ W	Terrestrial
Indian Grass (*Sorghastrum nutans*) Prairie	Homer, IL	40°05′39.1″ N; 87°99′65.4″ W	Terrestrial
Northwestern Illinois Agricultural Research and Demonstration Center	Monmouth, IL	40°93′64.6″ N; 90°72′06.5″ W	Terrestrial
Agricultural Field	Belleville, IL	38°31′59.5″ N; 89°53′40.2″ W	Terrestrial
Carpinteria Salt Marsh, Muddy Site	El Estero, CA	34°24′01.7″N; 119°32′21.6″ W	Marine
Carpinteria Salt Marsh, Sandy Site	El Estero, CA	34°23′55.6″ N; 119°32′19.3″ W	Marine
Carpinteria State Beach, Intertidal Site 1	Carpinteria, CA	34°23′35.4″ N; 119°31′30.4″ W	Marine
Carpinteria State Beach, Intertidal Site 2	Carpinteria, CA	34°23′35.4″ N; 119°31′30.4″ W	Marine

Abbreviation: DAPI, 4′,6‐diamidino‐2‐phenylindole.

Nematode extraction was accomplished through sieving and sugar centrifugation followed by the Baermann funnel method (MacGuidwin and Bender 2010). Nematodes were separated from sediment using 250 and 38‐μm pore size sieves. Material collected on a 38‐μm pore sieve was centrifuged at 3000 rpm for 3 min followed by sugar centrifugation at 3000 rpm for 3 min with 45% sucrose. The supernatant was applied to a 25‐μm pore size sieve for rinsing and collection. To prevent osmotic stress for marine nematodes, rinses were carried out with Instant Ocean® Sea Salt (Instant Ocean, Blacksburg, VA), prepared as per manufacturer's instructions.

To capture nematodes that might be missed by sieve extraction (e.g., those within roots or larger specimens), sediment collected on the 250‐μm sieve was processed using a Baermann funnel apparatus (MacGuidwin and Bender 2010). After 48 h, the sample was drained for collection. As with the sieve extraction steps, marine samples were steeped in Instant Ocean® mixture to ensure cellular preservation.

Nematodes were identified to the family level based on morphological keys (Abebe et al. 2006; Bongers 1988; Mai and Mullin 1996; Peña Santiago 2021; Schmidt‐Rhaesa and Rothe 2014).

### 2.2 4′,6‐Diamidino‐2‐phenylindole (DAPI) Staining and Ventral Nerve Cord Enumeration

Nematodes were centrifuged at 4000 rpm for 4 min in 15 mL Falcon tubes. The liquid was gently pipetted off and reduced to 1 mL. Nematodes isolated from aquatic environments tended to coil during the fixation step and, therefore, 50 μL of 0.1 M levamisole was added as a relaxant. Once adequately immobilized, all liquid except a thin layer was pipetted off and 1 mL of DESS (dimethyl sulfoxide, disodium EDTA, and saturated NaCl) was added (Yoder et al. 2006). For terrestrial samples, no sedative was added and all liquid except a thin layer was immediately pipetted off and 1 mL of saturated DESS was added and stored at 4°C. Depending on the amount of sediment in each sample, 2–8 μg/mL DAPI was added to the fixed specimen. The sample was kept in the dark at room temperature overnight and then returned to 4°C until imaging.

DAPI‐stained nematodes were mounted onto a 2% agar pad. Fluorescent microscopy was performed using an Axio Imager M2 fluorescent microscope equipped with an Axiocam 506 mono camera and an X‐Cite® Series 120Q fluorescent lamp (Zeiss, Oberkochen, Germany). Samples were screened for adults with consistent staining. Z‐stacks were taken with slices at 0.35‐0.5 μm intervals, collecting both the differential interference contrast (DIC) and DAPI channels for future reference. The entire nematode was imaged with sufficient overlap for subsequent stitching.

**Figure 2:**
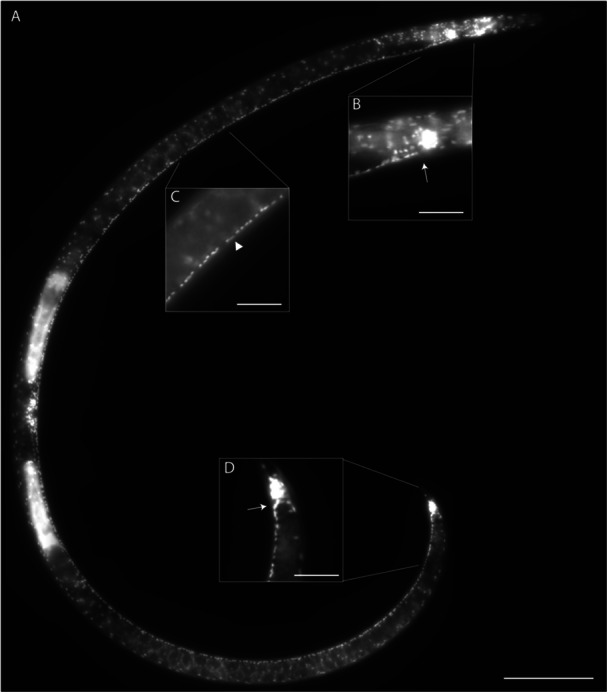
4′,6‐Diamidino‐2‐phenylindole (DAPI) staining of *Xiphinema* sp. (Dorylaimia, Clade 2). The ventral nerve cord (VNC) consists of a line of neurons extending along the ventral line from the retrovesicular ganglion (RVG) to the pre‐anal ganglion (PAG). (A) Lateral view of an adult female *Xiphenema* showing overall VNC structure. (B) Close‐up of the region at RVG and the anterior VNC (arrow). (C) Putative hypodermal (arrowhead) and adjacent neuronal nuclei of VNC. (D) Division between PAG and VNC (arrow). Scale bars = (A) 100 µm; (B–D) 25 µm.

DAPI‐stained nuclei were considered VNC neurons based on morphology, size, position, and relative intensity of stain, in accordance with previous work (Han et al. 2016; Sulston 1976; Sulston and Horvitz 1977; White et al. 1976). Neuronal nuclei in the VNC tend to be small, punctate, aligned, and brighter than surrounding nuclei (Figure 2b). Counts were conducted between the retrovesicular ganglion and the preanal ganglion immediately anterior to the anus using the multipoint tool in Fiji (Han et al. 2016; Schindelin et al. 2012). VNC length was measured from the nerve ring to the preanal ganglion. VNC neuron enumeration for each nematode was performed by two independent examiners (Supporting Information: Data 1). Although minor variations in counts were observed between the observers, the overall trends and statistical outcomes were consistent. Averages from the two observers are presented here (Figure 1). For *C. elegans*, 1 day old adult hermaphrodites of the N2 Bristol strain were used to determine VNC length. To account for the possible confounding effects of sex and developmental stage, we only examined adult females with exceptions for Camacolaimidae and Epsilonematidae. For Camacolaimidae, only males were found in our sampling, while for Epsilonematidae, males were included due to low sample numbers of females and consistency between sexes. Z‐stack image data is available at the Illinois Data Bank: https://doi.org/10.13012/B2IDB-6566418_V1.

### 2.3 Mononchus Aquaticus Culture

*M. aquaticus* was isolated from a maize field at the Northwestern Illinois Agricultural Research and Demonstration Center in Monmouth, Illinois and identified using morphological (Ahmad and Jairajpuri 2010) and 18S sequencing results (Holterman et al. 2006; GenBank Accession: PV282461). Our isogenic *M. aquaticus* culture was established from a single female cultured based on techniques described by Salinas (2004). Briefly, soil extract agar was made by adding water to 100 cm^3^ of soil up to a total volume of 1 L and incubating overnight at room temperature. The following day, the mixture was poured through a 25‐µm filter followed by centrifugation of the flow through solution for 3 min at 3000 rpm. The pellet was discarded and 500 mL of the supernatant was mixed with 5 g of agar and 500 µL of 5 mg/mL cholesterol dissolved in ethanol before autoclaving. Mixed stages of *Aphelenchus avenae*, originally isolated by Nathan Schroeder from Somerset, NJ and grown on half‐strength potato dextrose agar with *Monilinia fructicola* or *Botrytis cinerea*, were used as prey. *M. aquaticus* was fed *A. avenae* weekly or biweekly. No attempt was made to prevent microbial contamination. While *M. aquaticus* readily fed on *A. avenae*, we also observed juvenile *M. aquaticus* displaying pharyngeal pumping in the absence of a prey nematode, similar to observations made by Bilgrami (1984). *M. aquaticus* is considered parthenogenic and we have only observed a single male once.

### 2.4 Dye‐Filling Assay

In order to survey potential sensory neurons in *M. aquaticus*, we modified an established dye‐filling assay used in *C. elegans* (Tong and Bürglin 2010). A total of 37 *M. aquaticus* (21 juveniles and 16 adults) were subjected to a dye‐filling assay. Groups of five worms were placed in 1 mL of 5% M9 buffer and 2 µL of 2 mg/mL DiO stock solution. The nematodes were incubated for 5 h for adults or 4 h for juveniles. After incubation, nematodes were transferred to empty soil extract agar plates for a 30‐min recovery period. For imaging, anesthetization was achieved by applying 10 µL of 10 mM sodium azide solution onto a 5% agar pad. Images were captured using DIC and GFP filters on a Zeiss Axio Imager M2 microscope at 63× magnification. Z‐stack image data is available at the Illinois Data Bank: https://doi.org/10.13012/B2IDB-6566418_V1.

Image analysis was performed using Fiji (Schindelin et al. 2012). Neurons were counted in the head (from the tip of the head to immediately posterior to the nerve ring) and the body (from posterior of the nerve ring to the anus). Counts were conducted using the Cell Counter plugin in Fiji. The entire nematode length was measured in micrometers.

To ensure reproducibility and address potential errors due to the close apposition of nuclei in certain regions, enumeration was performed by three independent examiners. Although minor variations in counts were observed between the three observers (Supporting Information: Data 1), the overall trends and statistical outcomes were consistent, and we therefore averaged the counts for the final data used in this study. To determine the consistency of staining, we selected a defined 50 µm area surrounding the vulva on the left lateral side as a landmark and compared cell body position, number of processes and process directionality of body wall neurons within this region in multiple individuals. Only animals with bright staining in other parts of the nervous system were selected.

### 2.5 Bisection Assay

To test for movement despite severing of potential connections to the nerve ring, we developed a bisection assay. Twenty‐five adult female *M. aquaticus* and 25 adult hermaphrodite *C. elegans* were placed on microscopic slides and bisected with a 25‐gauge 5/8‐inch hypodermic syringe needle in 40–50 µL of distilled water for *M. aquaticus* or M9 buffer for *C. elegans*. Immediately after bisection, the number of tail bends was counted over 60 s and at 10‐, 20‐, and 30‐min post‐bisection. As a control, 25 uncut *M. aquaticus* and 25 uncut *C. elegans* worms were assessed for locomotion by counting body bends.

### 2.6 Data Analysis

#### 2.6.1 DAPI VNC Neuron‐Like Nuclei Analysis

VNC neuron‐like nuclei counts were analyzed using R (version 4.5.1) with the packages caper, phytools, nlme, emmeans, multcomp, MASS, dplyr, and ggplot2 (Orme et al. 2025; Revell 2012). Previously published counts for Chromadorian nematode species in Clades 9‐12 were included in the dataset (Han et al. 2016). For order‐level comparisons, groups with a sample size of 1 were excluded.

To determine the appropriate statistical framework, the phylogenetic signal (Pagel's λ; Pagel 1999) was initially assessed using phylogenetic generalized least squares (PGLS) on log‐transformed VNC counts (Freckleton et al. 2002; Martins and Hansen 1997). Diagnostic assessments using Shapiro‐Wilk and Q‐Q plots detected that the phylogenetic residuals deviated from normality. Consequently, alternative statistical methods were selected based on the estimated phylogenetic signal.

For subclass, where a strong phylogenetic signal was detected (*λ* = 1), phylogenetic ANOVA (Garland et al. 1993) with 1000 simulations were employed followed by post hoc pairwise comparison using the Holm method. For order‐level comparisons, where the phylogenetic signal was weak (*λ* = 0), a generalized linear model (GLM) was applied with a negative binomial distribution to the raw count data. Pairwise differences between orders were evaluated using estimated marginal means with Tukey's adjustment.

To examine the allometric relationship between VNC neuron count and VNC length, a phylogenetic ANCOVA was performed (Martins and Hansen 1997). Both VNC count and length were log‐transformed. Assumptions of normality and homoscedasticity of residuals were evaluated through Shapiro–Wilk, Q‐Q plots, and Breusch‐Pagan test. A PGLS model was fitted with subclass as a fixed factor and VNC length as a covariate. Model significance was evaluated using analysis of variance on the fitted PGLS model. Subsequently, post hoc pairwise comparisons of the estimated marginal means were performed with *p*‐value adjusted for multiple comparisons using Tukey.

#### 2.6.2 Dye‐Filled Neuron Analysis

For dye‐filling assays, a two‐sample t‐test was applied to compare differences in total neuron counts and head neuron counts between adults and juveniles using the t.test() function in R (version 4.5.1) and plotted using ggplot2 package. A simple linear regression was performed to examine the relationship between body length (µm) and dye‐filled neuron count for juvenile and adult separately as described above.

#### 2.6.3 Bisection Analysis

Repeated measures ANOVA was performed to compare body bends between time using tidyverse, rstatix R packages (R version 4.5.1). Time was the main factor and each subject was a repeated measures factor. Residuals were assessed for normality via the Shapiro–Wilk test and Q–Q plots.

## 3 Results

### 3.1 Total Number VNC Neuron‐Like Nuclei Does Not Support a One‐Time Simplification Process

**Figure 3:**
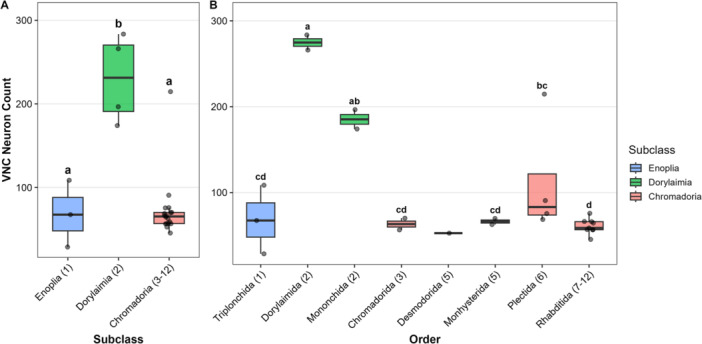
Comparison of ventral nerve cord (VNC) neuronal nuclei across subclasses (A) and orders (B). Each point represents the mean value of a family. Data from this study include adult females except for Camacolaimidae (Plectida, Clade 6) and Epsilonematidae (Desmodorida, Clade 5), which include males. Rhabditida, previously published in Han et al. (2016), included several taxa where juveniles were examined. Numbers in parentheses on the x‐axis indicate clade numbers (Holterman et al. 2006). (A) Subclass differences were tested using phylogenetic analysis of variance (ANOVA) with 1000 simulations (*p* = 0.003). Different letters denote statistically significant differences by Holm's post hoc test at *α* = 0.05. (B) Order differences were tested using a generalized linear model (*p* < 2.2e−16). Different letters denote statistically significant differences by Tukey's post hoc test at *α* = 0.05. Numbers in parentheses on the x‐axis indicate clade numbers (Holterman et al. 2006). [Color figure can be viewed at wileyonlinelibrary.com]

Similar to our previous work (Han et al. 2016), we used nuclear morphology as an indicator of cell type in order to enumerate putative neurons in the VNC of multiple nematode species. However, as few species in early diverging lineages are considered culturable, we made our observations on nematodes directly recovered from diverse habitats (Table 1). Individuals were identified to the family level and the number of VNC neuron‐like nuclei were counted between the retrovesicular ganglion and pre‐anal ganglion (Figure 2a–d). VNC neuron data were compared across subclass and order. We found significant differences among subclasses (Figure 3a; *p* = 0.003). Specifically, Dorylaimia (Clade 2) exhibited significantly higher number of neuronal nuclei compared to Enoplia (Clade 1) and Chromadoria (Clades 3–12). VNC neuronal nuclei counts also differed significantly among orders (*p* < 2.2e−16; Figure 3b). Dorylaimida (Clade 2) showed significant higher numbers compared with other orders in Enoplia and Chromadoria. Mononchida (Clade 2) also had higher numbers than others except Plectida (Clade 6). While many families in Plectida had higher VNC neuronal nuclei than those in Rhabditida (Clades 8–12), Camacolaimidae from marine samples was an obvious outlier and likely contributed disportionally to the statistical difference found between Plectida and Rhabditida (Figure 3b).

**Figure 4:**
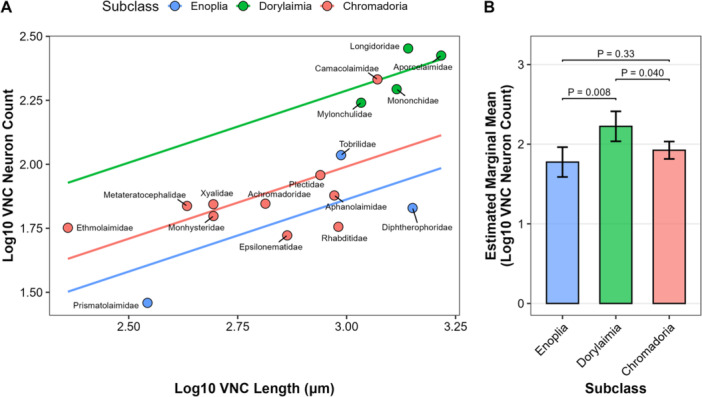
Phylogenetic analysis of covariance (ANCOVA) of ventral nerve cord (VNC) neuronal nuclei counts across subclasses. (A) Scatter plot depicting the relationship between log‐transformed VNC length and log‐transformed VNC neuronal nuclei counts. Each point represents the mean value of a family. Solid lines indicate the fit of a phylogenetic ANCOVA model assuming a common slope for all subclasses (slope = 0.56, adjusted *R* ^2^ = 0.75), with phylogenetic signal *λ* estimated at 0. The model indicates a significant positive association between VNC length and neuronal nuclei counts (*F*_1,13_ = 35.50, *p* < 0.001) and significant differences in intercepts among subclasses (*F*_2,13_ = 7.59, *p* = 0.007). (B) Post hoc pairwise comparisons of estimated marginal means of log‐transformed VNC neuronal nuclei counts among subclasses, adjusted for VNC length. Error bars represent 95% confidence intervals. *p*‐values were adjusted using Tukey's method. [Color figure can be viewed at wileyonlinelibrary.com]

Despite having hugely different body sizes and numbers of muscle cells in their adult stages, *C. elegans* (Clade 9) and *A. suum* (Clade 8) have nearly identical numbers of VNC motor neurons (Stretton et al. 1978), suggesting that number of neurons does not necessarily need to scale with body size. However, we wanted to determine if there was a relationship between body size and VNC neurons outside of the Chromadoria. To disentangle the effect of body size from taxonomic differences, we performed phylogenetic ANCOVA with VNC length as a covariate (Figure 4a). The analysis revealed a strong positive association between VNC length and number of neuronal nuclei (*R* ^2^ = 0.80, *F*_1,13_ = 34.77, *p* < 0.001). The subclasses differ significantly in their intercepts of neuronal nuclei counts (*F*_2,13_ = 8.72, *p* = 0.004), indicating grade shifts in neuron numbers at a given body length. Dorylaimia possessed a significantly higher number of VNC neuronal nuclei compared to both Enoplia (*p* = 0.008) and Chromadoria (*p* = 0.04) after controlling for VNC length (Figure 4b). In contrast, no significant difference was found between Chromadoria and Enoplia (*p* = 0.33).

Together, these results do not support a one‐time simplification of neuroanatomy. While there is a general trend of VNC neuronal numbers scaling with body size, Dorylaimia shows significantly higher baseline neuronal numbers compared to other groups.

### 3.2 *Mononchus aquaticus* Exhibits Determinate‐Like VNC Development

**Figure 5:**
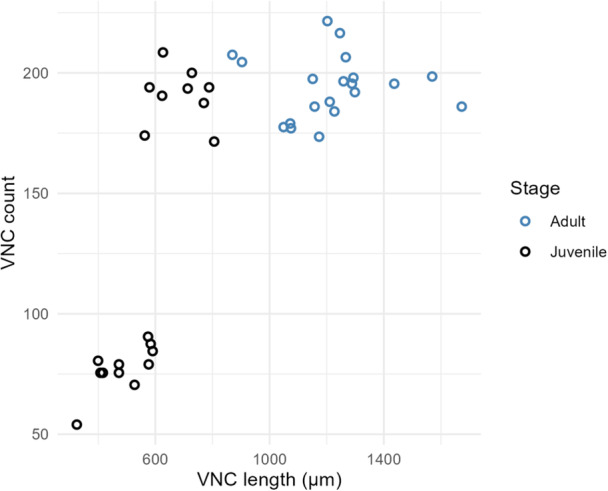
Ventral nerve cord (VNC) neuronal nuclei counts through the development of cultured *Mononchus aquaticus*. A positive association between VNC neuronal nuclei counts and VNC length (µm) was found in juveniles (*p* < 0.001, adjusted *R* ^2^: 0.59, slope: 0.33), while no association was found in adults (*p* = 0.996, adjusted *R* ^2^: −0.06, slope: −7.7 × 10^−5^). Black circles represent juveniles; blue circles represent adults. [Color figure can be viewed at wileyonlinelibrary.com]

Our survey data suggest that the Dorylaimia had more VNC neuronal nuclei than other taxa in our survey (Figure 3a). However, these data could not address intraspecific variability. Within Dorylaimia, previous studies have reported the successful cultivation of Mononchida nematodes (Grootaert and Maertens 1976; Maertens 1975; Nelmes 1974; Salinas 2004). Mononchida comprises predatory nematodes characterized by teeth. To better examine nervous system development and intraspecific variability, we established an isogenic culture of the likely parthenogenic species *Mononchus aquaticus* from a single female. We counted 198 ± 12 (*n* = 20) neuronal nuclei in the VNC of adult *M. aquaticus* females (Figure 5).

In *C. elegans*, only a subset of VNC neurons form during embryogenesis. This is followed by the rapid addition of additional neurons and rewiring during late L1 (Sulston 1976). Similar developmental timelines were found in other Chromadoria (Bui and Schroeder 2018; Han et al. 2016). We wished to see if a similar process occurs within *M. aquaticus*. As we have not strictly determined the developmental timeline, we used VNC length as a surrogate for developmental stage. Our results suggest a similar pattern of VNC development as found in *C. elegans* (Sulston and Horvitz 1977). The smallest juveniles (presumed J1s, as most free‐living nematodes hatch at this stage, though molts were not tracked in this study) had counts of 54–94 VNC neuron‐like nuclei (Figure 5). However, this number increased rapidly during juvenile development to approximately 200 VNC neuron‐like nuclei. No correlation between VNC length and number of neuron‐like nuclei was found following this rapid increase.

### 3.3 Dye‐Filling Reveals Expanded Neuron Distribution in *M. aquaticus*

The substantially higher VNC neuron counts observed in *M. aquaticus* raised a question of whether this expansion extends to other parts of the nervous system. To investigate sensory neurons in *M. aquaticus*, we performed dye‐filling assays, which selectively label sensory neurons with ciliated endings exposed to the environment in *C. elegans* (Tong and Bürglin 2010). In wild‐type *C. elegans*, dye‐filling consistently labels six pairs of amphid neurons in the head, two pairs of phasmid neurons in the tail, and six inner‐labial 2 (IL2) neurons depending on salt concentration (Tong and Bürglin 2010). While some variation exists, dye‐filling among other Chromadorian nematodes is restricted to neurons in the anterior and posterior ends (Garg et al. 2022; Han et al. 2016; Srinivasan et al. 2008).

**Figure 6:**
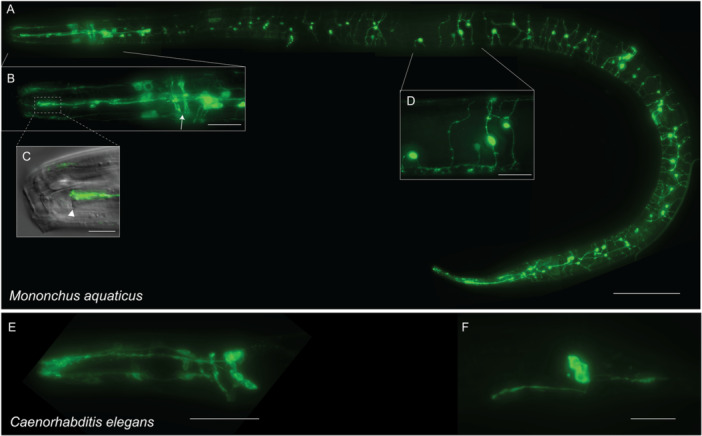
Dye‐filling in adult *Mononchus aquaticus* (A–D) and *Caenorhabditis elegans* (E, F). (A) Lateral view of entire body of dye‐filled female *M. aquaticus*. (B) Dye‐filled head sensory neuron cell bodies and the nerve ring (arrow). Putative inner labial and amphid neuron dendrites extend to the anterior end. The nerve ring appears as two separate bundles and receives processes from surrounding neurons. (C) Amphid neuron dendrites terminate at the slit‐shaped amphid aperture (arrowhead). (D) Body wall neurons are distributed throughout the body, with processes extending to the ventral and dorsal nerve cords to form commissures. (E, F) Dye‐filling in L3 *C. elegans*. Unlike *M. aquaticus*, no body wall neurons are dye‐filled in *C. elegans*. (E) Dye‐filled inner labial and amphid in the head. (F) Dye‐filled phasmid neurons in the tail. Scale bars = (A) 100 µm; (C) 10 µm; (B, D) 25 µm; (E, F) 20 µm. [Color figure can be viewed at wileyonlinelibrary.com]

**Figure 7:**
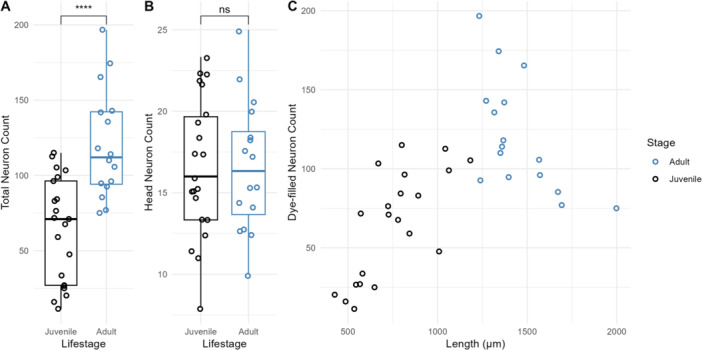
Ontogeny of dye‐filled neuron number in *Mononchus aquaticus* juveniles and adults. (A) Total dye‐filled neuron counts in adults versus juveniles. Adults have more dye‐filled neurons (120 ± 36, *n* = 16) compared to juveniles (64 ± 35, *n* = 21; *p* < 0.0001, *t*‐test). (B) Dye‐filled head neuron counts in adults (17 ± 4, *n* = 16) versus juveniles (16 ± 4, *n* = 21). No significant difference was detected (*p* = 0.9966, *t*‐test). (C) Relationship between total dye‐filled neuron count and body length (µm) in *M. aquaticus*. While there is a moderately positive association of dye‐filled neuron counts and body length in juveniles (*p* < 0.001, adjusted *R* ^2^: 0.498, slope: 0.121), a weak negative association was found in adults (*p* < 0.011, adjusted *R* ^2^: 0.330, slope: −0.108). [Color figure can be viewed at wileyonlinelibrary.com]

In contrast to this restricted pattern, *M. aquaticus* showed a substantial expansion in dye‐filling pattern (Figure 6a–d). We observed anterior neurons similar in position and morphology to the amphid and IL neurons of *C. elegans*, although staining of the circumpharyngeal nerve ring appeared as two distinct anterior and posterior regions (Figure 6a,b). The most conspicuous difference in dye‐filling between *M. aquaticus* and *C. elegans* was the presence of numerous dye‐filled neurons distributed along the entire length of the *M. aquaticus* lateral body walls (Figure 6a,d), which is a feature absent in all previously examined Chromadorian species (Garg et al. 2022; Han et al. 2016; Srinivasan et al. 2008; Tong and Bürglin 2010). In total, we counted 120 ± 36 (*n* = 16) dye‐filled neurons in *M. aquaticus* adults (Figure 7a), in contrast to the 22 neurons that typically dye fill in *C. elegans* (Figure 6e,f).

**Figure 8:**
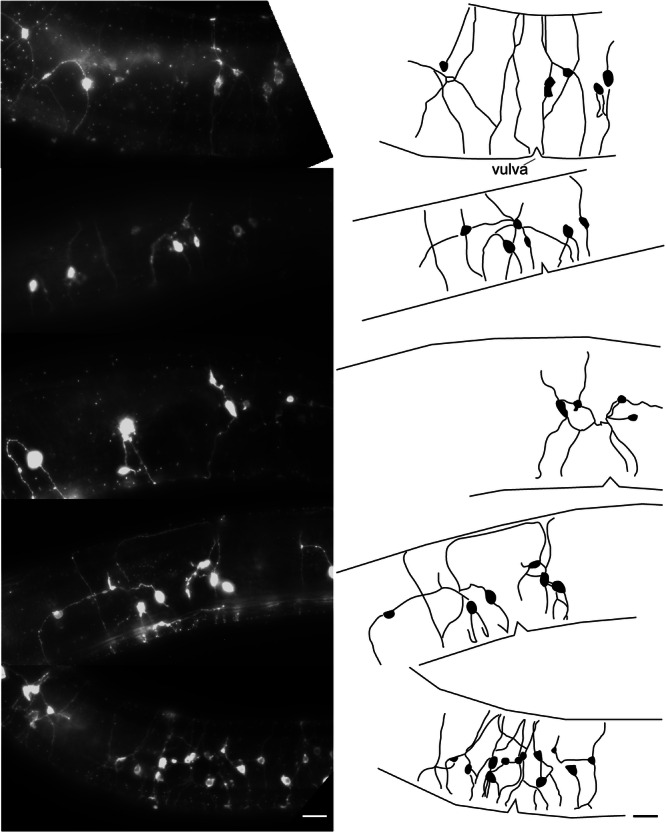
Lateral view images of dye‐filled neurons near the vulva in adult *Mononchus aquaticus* females (left), showing the arrangement of neuronal cell bodies and processes. All nematodes are oriented with anterior to the left and the dorsal side up. (right) Corresponding schematic outlines of neurons (black fill) and processes adjacent to the vulva (shown as triangular notch). Processes within the ventral or dorsal cords were not drawn as it was impossible to differentiate individual neuronal processes within the cords. Scale bar = 10 µm.

The nervous system of *C. elegans* is highly stereotyped. While many *C. elegans* neuron cell bodies show some positional variability, this is typically on the order of 10 µm or less (Toyoshima et al. 2020; Yemini et al. 2021). In contrast, we observed considerable inter‐individual variability in *M. aquaticus* body wall neurons within a defined region (Figure 8). Each animal displayed a distinct number of neurons and branching pattern. The large amount of variation in dye‐filling counts observed in *M. aquaticus* could be due to both variation in uptake of dye by individual neurons and differences in the number of neurons between individuals. Inter‐individual variability in body wall neuron number has been reported in other Enoplid nematode species (Malakhov 1982).

The specific morphology of the body wall neurons varied. All body wall neurons included at least two processes emerging from the cell body; however, many neurons included three or four processes. In all instances, at least one process traveled to the VNC. Occasionally, both processes of bipolar neurons entered the VNC. We also observed occasional commissures where neuronal processes traveled from the dorsal to ventral cords without an apparent cell body (Figure 6d). As we never observed dye‐filled cell bodies within the VNC or dorsal cord (DC), these commissures likely originated from a cell body in the lateral body wall that sent a process into the VNC or DC, which then extended back to the opposite cord. While most body wall neurons were separate enough to discern individual cell bodies and processes, we also regularly observed clusters of four to five body wall neurons in close enough proximity that we could not determine the source of individual processes.

Next, we examined whether sensory neuron numbers change during development. In *C. elegans*, the dye‐filling pattern is consistent across post‐embryonic development. Similar to VNC neuron counts, we found more dye‐filled neurons in adult *M. aquaticus* compared to juveniles (Figure 7a). Interestingly, we found that the head sensory neuron (putative amphid and IL neurons) numbers remained consistent throughout development (Figure 7b), suggesting that head sensory neurons may form during embryogenesis while body sensory neurons are continuously added during post‐embryonic development as the nematode increases in length. When examined as a function of total body length, juvenile dye‐filled neurons in *M. aquaticus* correlated positively with body length (*p* < 0.001, adjusted *R* ^2^: 0.498, slope: 0.121) but plateaued in adults (*p* < 0.011, adjusted *R* ^2^: 0.330, slope: −0.108) (Figure 7c). Unlike the developmental timeline of the VNC, which showed a one‐time rapid increase in the number of neuronal nuclei (Figure 5), dye‐filled neurons showed a gradual addition during juvenile development.

### 3.4 Decentralized Post‐Bisection Response in *M. aquaticus*

**Figure 9:**
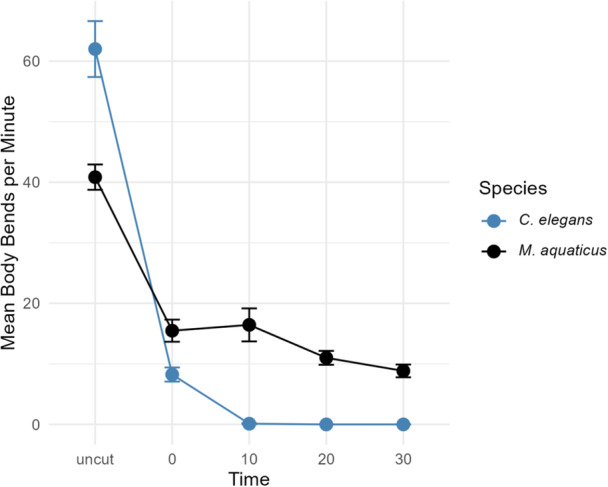
Temporal profile of body bends per minute prior to and following bisection in *Caenorhabditis elegans* (blue) and *Mononchus aquaticus* (black). Measurements were taken for uncut animals and subsequent time points (0, 10, 20, and 30 min post bisection). Error bars represent the standard error of the mean. [Color figure can be viewed at wileyonlinelibrary.com]

We hypothesized that the increased number of neurons in *M. aquaticus* compared to *C. elegans* will lead to changes in behavior. Anecdotally, we did not notice obvious behavioral novelties in *M. aquaticus*. However, during preparation of specimens for molecular identification, we observed that bisected *M. aquaticus* individuals unexpectedly continued to move rapidly for a prolonged period, which is not reported in other nematode species. To test this further, we compared the body bends in the posterior halves of *M. aquaticus* and *C. elegans* before and after bisection (Figure 9). Intact and live *C. elegans* and *M. aquaticus* showed 62 and 40.8 body bends per minute, respectively. Following bisection, *C. elegans* showed an immediate reduction to 8.2 bends per minute, with near‐complete cessation by 10 min post‐bisection. In contrast, *M. aquaticus* maintained reduced but persistent body bending, showing gradual decline over 30 min postbisection. These results may suggest a more decentralized nervous system in *M. aquaticus*.

## 4 Discussion

**Figure 10:**
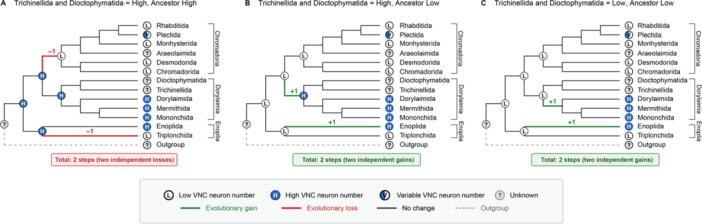
Parsimony reconstruction of ancestral VNC neuron number in Nematoda. Three alternative evolutionary scenarios based on the unknown state of *Trichonellida* and *Dioctophymatida*. Enoplida is assumed to have high VNC neuron numbers based on the previous report (Malakhov 1994). (A, B) If *Trichonellida* and *Dioctophymatida* have high VNC neuron numbers, two equally parsimonious reconstructions exist: (A) two independent losses from a high ancestral state or (B) two independent gains from a low ancestral state. (C) If *Trichonellida* and *Dioctophymatida* have low VNC neuron numbers, parsimony supports two independent gains from a low ancestral state. Node circles indicate hypothetical ancestral states; tip circles indicate observed or hypothesized (*Trichonellida* and *Dioctophymatida*) states. Edge colors denote evolutionary transitions: green = gain, red = loss, black = no change, dashed gray = outgroup. H, high VNC neuron number; L, low VNC neuron number; V, variable within order; ?, unknown state. [Color figure can be viewed at wileyonlinelibrary.com]

As we found significantly more VNC neuronal nuclei on average in Dorylaimia (Clade 2) compared with Enoplia (Clade 1) and Chromadoria (Clades 3–12), our analysis of VNC neuronal nuclei did not support the hypothesis of a one‐time secondary simplification of the nervous system during nematode evolution. Instead, our findings suggest alternative scenarios, each with multiple evolutionary changes in number of neurons (Figure 10). Several pieces of data prompted our original hypothesis of a reduction in the number of neurons occurring during the split of Chromodoria from Enoplia and Dorylaimia. Our previous data and others showed that no species in Clades 9–12 had more than 76 VNC neurons (Han et al. 2016). This contrasted with the hundreds to thousands of neurons reported in Enoplian or Dorylaimian nematodes *Pontonema vulgare* (Clade 1, Enoplia; Malakhov 1994), *Mermis nigrescens* (Clade 2, Dorylaimia; Gans and Burr 1994), and *Longidorus macrosoma* (Clade 2, Dorylaimia; Sulston and Horvitz 1977). Indeed, in his comprehensive book on nematode structure, Malakhov 1994 suggested a general decrease in the number of neurons between Enoplida (Clade 1) and Rhabditida. A similar suggestion was made regarding the number of sensory neurons between the paraphyletic class Adenophorea (Clades 1–6) and Secernentea (Clades 8–12) (Coomans and De Grisse 1981). However, in our dataset, both Enoplia (represented by Triplonchida) and Chromadoria have a lower number of VNC neuronal nuclei than Dorylaimia. Additionally, within the Chromadoria, the Camacolaimidae (Clade 6, Plectida) appears as an outlier with over 200 putative VNC neuronal nuclei (Figures 1, 3, 4). Our VNC counts are based on DAPI‐stained nuclei to identify neurons, which introduces two potential sources of error. First, our VNC counts do not include possible neurons that lie within the preanal or retrovesicular ganglia. In *C. elegans*, 10 motor neurons that are logically part of the VNC are found within the retrovesicular ganglion (White et al. 1976). The distribution of homologous neurons in other species is unknown. Second, it is possible that neuronal‐like DAPI staining patterns are not representative of neurons in other species or vice versa that non‐neuronal DAPI staining patterns are neurons. Lower throughput methods based on electron microscopy or immunohistochemistry may be required to confirm our findings. However, we observed variability in nuclear morphology similar to that observed in *C. elegans*, strongly suggesting our counts are generally valid and reflect genuine biological differences.

Given the consensus phylogeny in which Enoplia represents the earliest‐diverging nematode lineage, followed by Dorylaimia, and the Chromadoria (Holterman et al. 2006; Qing et al. 2024), the most parsimonious reconstructions among major nematode orders require two evolutionary changes, though the specific scenario depends on the states of unsampled lineages (Figure 10). It is important to note that our current dataset lacks representatives from Enoplida (Clade 1, Enoplia), Trichinellida and Dioctophymatida (Clade 2, Dorylaimia), which introduces uncertainty into the ancestral state reconstruction. We assume that Enoplida has high VNC neuron numbers in our model based on previous reports in marine species (Malakhov 1994). In contrast, no VNC observations have been reported for Trichinellida and Dioctophymatida, thus the most parsimonious scenario depends on their unknown states. If Trichinellida and Dioctophymatida possess high VNC neuron numbers comparable to the rest of Dorylaimia, parsimony would yield two equally probable reconstructions: (1) an ancestral high‐neuron state with two independent losses in Triplonchida and Chromadoria (Figure 10a), (2) an ancestral low‐neuron state with two independent gains in both Enoplida and Dorylaimia (Figure 10b). If Trichinellida and Dioctophymatida possess low VNC neuron numbers similar to Triplonchida and most of Chromadoria, parsimony supports an ancestral low‐neuron state with two independent gains in Enoplida and Dorylaimia (Figure 10c).

Of particular interest, Camacolaimidae (Clade 6) represents an exception within Chromadoria. Within Plectida, Camacolaimidae is a derived family, whereas the remaining three families (Aphanolaimidae, Metateratocephalidae, and Plectidae) show low neuron numbers. This suggests that the common ancestor of Plectida possessed low VNC neuron numbers and the elevated counts in Camacolaimidae represent an independent gain that occurred regardless of the large‐scale evolutionary changes at deeper nodes. Thus, all scenarios require at least two evolutionary steps and a secondary increase in neuron number for the Camacolaimidae.

The relationship between habitat and neuronal number remains an open question. We were only able to recover, stain, and identify two marine taxa from Epsilonematidae (Clade 4) and Camacolaimidae. The Camacolaimidae comprises a group of marine species that had notably more neurons than other basal Chromadoria taxa. While this, together with the work by Malakhov (1994), may suggest that increased numbers of neurons are adaptive for marine life, our counts from the Epsilonematidae do not support this hypothesis. Certain taxa have evidently experienced unknown evolutionary pressures, resulting in exceptional length‐to‐neuron ratios. Future studies incorporating additional Enoplida, Trichinellida, and Dioctophymatida species, along with potential outgroup comparisons to sister phyla such as Nematomorpha and Tardigrada (Qing et al. 2024; Smythe et al. 2019), will resolve the ancestral state of the nematode nervous system with greater confidence.

Despite having four times more VNC neurons, *M. aquaticus* shares similar post‐embryonic VNC developmental timeline as Chromadorian species. For example, *C. elegans* P‐blast cells undergo a fixed program of division during late L1 to quickly generate the final complement of VNC motor neurons (Sulston 1976). This determinate pattern of VNC development has been observed in several other Chromadorian species (Han et al. 2016). Previous studies on nematode embryonic development have reported that certain species in Enoplia and Dorylaimia (including Mononchida) display high variability in early cleavage patterns and cell fate determination (Malakhov 1994; Schulze and Schierenberg 2011). Based on these observations, nematode development has been proposed to have evolved from an indeterminate and variable mode characteristic of Enoplia toward the determinate mode seen in derived Chromadorian clades (Schulze et al. 2012; Schulze and Schierenberg 2011). However, whether these differences in early embryonic development have direct consequences for post‐embryonic neural development has not been examined in Enoplia or Dorylaimia. The determinate‐like VNC developmental pattern observed in *M. aquaticus* in the present study suggests that, despite variability in early embryogenesis, the determinate nature of post‐embryonic VNC development may have been present at or before the divergence of Dorylaimia.

The dye‐filling assay revealed that *M. aquaticus* possess extensive body wall neurons that are absent in *C. elegans* and other Chromadorian species (Han et al. 2016; Hong et al. 2019; Tong and Bürglin 2010; White et al. 1986). This finding suggests that the loss of body wall sensory neurons may represent a derived feature in Chromadoria. The most similar neuron classes in *C. elegans* are the two pairs of anterior and posterior deirid neurons; however, the deirid ciliated endings are not exposed to the environment and do not dye‐fill in wild‐type animals (Perkins et al. 1986). In contrast, numerous body wall neurons with ciliated endings have been reported from several Enoplian and Dorylaimian species. In marine Enoplian species, these neurons have been described as proprioceptors (Hope and Gardiner 1982) or associated with setae (Malakhov 1994). In Dorylaimia, *Xiphinema americanum* (Clade 2) has ciliated sensory neurons innervating body pores that allow for chemoreception (Wright and Carter 1980). The *M. aquaticus* body wall neurons may be associated with body pores, analogous to those in *X. americanum* (Wright and Carter 1980). An ultrastructural examination of the body wall neurons in *M. aquaticus* will be necessary to determine their homology and possible functional role.

Our finding that *M. aquaticus* can continue movement long after they have been bisected from connection to the central nerve ring may suggest a more distributed motor control system compared to the centralized control of *C. elegans*. One possible explanation is that the extensively distributed body wall neurons contribute to this response. These neurons project directly to the VNC and dorsal cord, which could enable local sensory inputs to drive motor output independently of the nerve ring. An alternative explanation is that *M. aquaticus* is better able to maintain cellular integrity following a traumatic event. Additional physiological and connectivity analyses will be necessary to test these hypotheses.

The increased VNC neuron number in *M. aquaticus* does not appear to correlate with increased speed or other novel locomotory behavior in intact individuals. Furthermore, the predatory behavior of *M. aquaticus* is unlikely to account for the increased neuron number as *P. pacificus*, which is a facultative predator more closely relative to *C. elegans*, does not display an increase in number of neurons. Indeed, the Dorylaimia specimens in our survey include diverse feeding habits such as omnivorous (Aporcelaimidae), predatory (Mononchidae and Mylonchulidae), and plant‐parasitic (Longidoridae), yet all possess high VNC neuron counts. We speculate that the decentralized control seen here reflects an ancestral condition that persists in early‐diverging nematodes rather than an adaptation to specific ecological demands.

## 5 Conclusions

Contrary to the hypothesis of a one‐time evolutionary simplification, our results indicate multiple phylum‐wide evolutionary changes in nematode VNC neuron numbers. Our established isogenic line of *M. aquaticus* showed that determinate‐like VNC development, seen in derived Chromadorian species, may have been present at or before the divergence of Dorylaimia. Notably, *M. aquaticus* also demonstrated extensive sensory neuron distribution in the head and along its body length, which is distinct from the restricted dye‐filling of head sensory neurons observed in more recently derived taxa. Bisection assays using *M. aquaticus* further suggest that basal nematodes may possess a less centralized nervous system.

## Conflicts of Interest

The authors declare no conflicts of interest.

## Supporting information


**Supporting Data 1:** Raw data for VNC neuron counts, dye‐filling assays, and bisection experiments.

## Data Availability

The data that support the findings of this study are openly available in Illinois Data Bank at https://databank.illinois.edu/, reference number https://doi.org/10.13012/B2IDB-6566418_V1.
